# Targeting chromatin modifying complexes in acute myeloid leukemia

**DOI:** 10.1093/stcltm/szae089

**Published:** 2024-11-28

**Authors:** Alexandra Schurer, Shira G Glushakow-Smith, Kira Gritsman

**Affiliations:** Department of Cell Biology, Albert Einstein College of Medicine, Bronx, NY 10461, United States; The Ruth L. and David S. Gottesman Institute for Stem Cell Research and Regenerative Medicine, Albert Einstein College of Medicine, Bronx, NY 10461, United States; Department of Cell Biology, Albert Einstein College of Medicine, Bronx, NY 10461, United States; The Ruth L. and David S. Gottesman Institute for Stem Cell Research and Regenerative Medicine, Albert Einstein College of Medicine, Bronx, NY 10461, United States; Department of Cell Biology, Albert Einstein College of Medicine, Bronx, NY 10461, United States; The Ruth L. and David S. Gottesman Institute for Stem Cell Research and Regenerative Medicine, Albert Einstein College of Medicine, Bronx, NY 10461, United States; Department of Medical Oncology, Albert Einstein College of Medicine, Montefiore Medical Center, Bronx, NY 10461, United States; Center for Tumor Dormancy, Montefiore Einstein Comprehensive Cancer Center, Albert Einstein College of Medicine, Bronx, NY 10461,United States; Marilyn and Stanley M. Katz Institute for Immunotherapy for Cancer and Inflammatory Disorders, Albert Einstein College of Medicine, Bronx, NY 10461, United States

**Keywords:** acute myeloid leukemia, chromatin, histones, KMT2A, PRC1, PRC2, *HOXA/B*, *MEIS1*, KMT2A-rearrangement, NPM1c, menin inhibition

## Abstract

Acute myeloid leukemia (AML) is a devastating hematologic malignancy with high rates of relapse, which can, in part, be attributed to the dysregulation of chromatin modifications. These epigenetic modifications can affect the capacity of hematopoietic cells to self-renew or differentiate, which can lead to transformation. Aberrant histone modifications contribute to the derepression of self-renewal genes such as *HOXA/B* and *MEIS1* in committed hematopoietic progenitors, which is considered a key mechanism of leukemogenesis in MLL-rearranged (MLL-r) and NPM1-mutated AML. As regulators of some of the key histone modifications in this disease, the menin-KMT2A and polycomb repressive (PRC1/2) complexes have been identified as promising targets for the treatment of AML. This review explores recent discoveries of how leukemic cells hijack these complexes and their interactions with other chromatin regulators to promote disease progression. We also discuss inhibitors targeting these complexes that have demonstrated therapeutic efficacy in preclinical and clinical studies and propose novel therapeutic combinations targeting the KMT2A and PRC1/2 broader interacting networks to overcome issues of resistance to existing monotherapies.

## Graphical Abstract

**Graphical Abstract:**
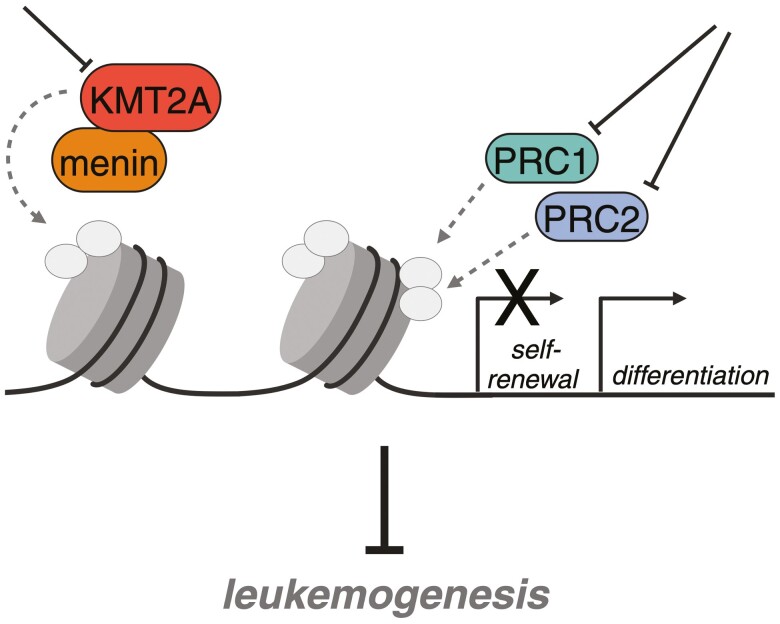

Dysregulation of the menin-KMT2A and polycomb repressive (PRC1/2) complexes in MLL-rearranged and NPM1-mutated acute myeloid leukemia (AML) contributes to the aberrant expression of self-renewal genes in hematopoietic stem and progenitor cells. Inhibitors targeting these complexes impair leukemic cell self-renewal and promote differentiation to hinder AML disease progression.

**Significance Statement**

Dysregulation of chromatin modifiers such as the menin-KMT2A complex and the polycomb repressive complex (PRC1/2) has been implicated in the pathogenesis of acute myeloid leukemia as well as in disease relapse. This review summarizes our current understanding of how these complexes dysregulate gene expression through histone modifications and provides a rationale for novel strategies to improve the clinical outcomes of existing therapies that target chromatin modifiers in AML.

## Introduction

Epigenetic marks are critical for allowing cells which contain the same genome to have varying functions by regulating gene expression in a cell-specific manner. These marks include both marks directly deposited on DNA, as well as marks on histones, the proteins involved in organizing DNA. The evolution of targeting aberrant DNA methylation in acute myeloid leukemia (AML) has been extensively reviewed.^1-5^ Therefore, this review primarily focuses on the regulation of chromatin accessibility and gene expression through histone modifications in AML. Specifically, we contextualize recent advancements in the mechanistic understanding of how leukemic cells hijack and dysregulate the KMT2A-related network and polycomb repressive complex 2 through mutations or chromosomal rearrangements as well as potential therapeutic approaches that have been tested preclinically or clinically to target the resulting chromatin alterations. We also discuss how combination therapies can capitalize on interactions within this network to overcome resistance and increase sensitivity to existing therapies that target histone modifiers in AML.

### MLL-rearranged acute leukemias

KMT2A, also known as MLL1, is a large, multi-domain histone methyltransferase involved in the positive regulation of transcription of several genes. This includes the *HOX* cluster genes, which are critically important for tissue differentiation during embryogenesis and for stem cell self-renewal during hematopoiesis.^6-8^ The C-terminal SET (Su(var.)3-9, enhancer of zeste and trithorax) domain of KMT2A is responsible for the deposition of transcription-activating di- and tri-methylation marks on histone H3 on lysine residue 4 (H3K4me2/3). The N-terminal AT-hook domains and a CxxC zinc-finger domain enable direct binding of KMT2A to its target genes.^6,8,9^ KMT2A can also indirectly associate with chromatin through N-terminal interactions with other DNA-binding proteins. KMT2A recruits the tumor suppressor menin, which functions as an adaptor to link KMT2A with the transcriptional co-activator LEDGF that directly binds promoter regions of KMT2A target genes. For a subset of KMT2A target genes, such as *HOXA9* and *MEIS1*, an association of menin with the promoter is required for H3K4 methylation and activation of gene expression.^6,9,10^ As an activator of *HOX* gene expression, wildtype KMT2A plays a critical role in regulating the self-renewal of hematopoietic stem cells (HSCs) during normal hematopoiesis.^7,11^

*KMT2A* is frequently involved in translocations at chromosome 11q23, resulting in the generation of oncogenic KMT2A fusion proteins (KMT2A-FP). These KMT2A rearrangements are responsible for ~75% of acute leukemias (AML, acute lymphoblastic leukemia/ALL, and mixed-lineage leukemia/MLL) in infants and up to 10% in children and adults, and are also found in therapy-related AML.^8,12^ During KMT2A rearrangement, the C-terminal SET domain is replaced with one of several different fusion partner proteins, which are most frequently transcription co-factor proteins such as AFF1 (AF4), MLLT3 (AF9), MLLT10 (AF10), MLLT1 (ENL), or ELL. The retained N-terminal segment of KMT2A directs the fusion proteins to KMT2A target genes.^8,12,13^ Despite the loss of the catalytic domain that is responsible for H3K4 methylation, most KMT2A fusions still cause over-expression of *HOXA* genes and their co-factors *MEIS1* and *PBX3* in both HSCs and committed progenitors to promote aberrant self-renewal, proliferation, and leukemogenesis.^14,15^

**Figure 1.**
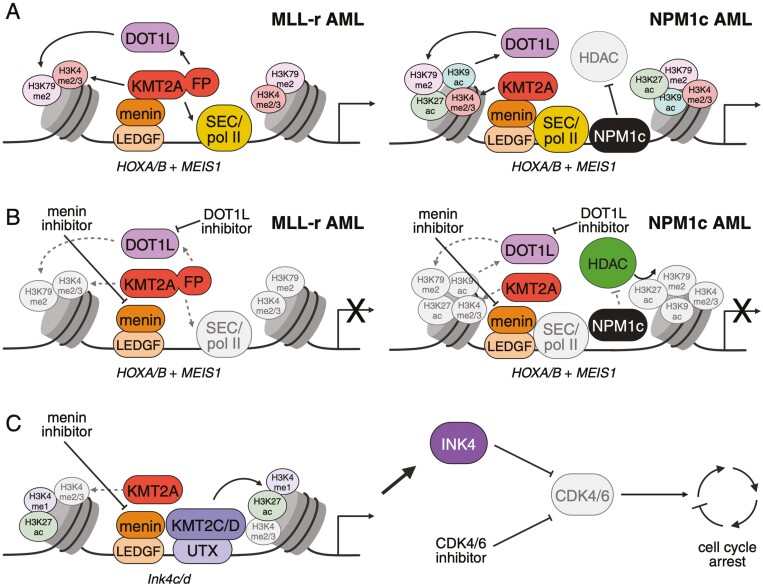
(A) The extended KMT2A network regulates histone modifications and chromatin accessibility to aberrantly activate transcription of HOXA/B cluster and MEIS1 target genes in MLL-rearranged (MLL-r) and NPM1-mutated (NPM1c) acute myeloid leukemia (AML). Menin inhibitors may synergize with (B) DOT1L inhibitors and (C) CDK4/6 inhibitors to treat MLL-r and NPM1c AML. Figure created with BioRender.com

KMT2A-FPs form large multiprotein complexes to activate leukemogenic gene expression through several mechanisms, which can be categorized as (1) transcriptional initiation and elongation and (2) chromatin remodeling and histone modifications (Figure 1A). Under physiological conditions, AFF1, MLLT1, and ELL associate with pTEFb (Positive Transcription Elongation Factor b) to form the Super Elongation Complex (SEC), which releases RNA Polymerase II (Pol II) from pausing to facilitate transcriptional elongation.^16,17^ In the context of acute leukemia, KMT2A-AFF1 and KMT2A-MLLT1 fusion proteins inappropriately recruit other components of the SEC to MLL target genes to promote and sustain the transcription of leukemogenic self-renewal genes such as *HOXA9*.^17,18^ MLLT3, one of the most common fusion proteins for KMT2A, similarly acts as a transcriptional activator both indirectly through recruitment of the SEC and directly through maintaining Pol II elongation. Loss of KMT2A-MLLT3 binding to the promoter regions of *HOXA9* and *MEIS1* results in pausing and loss of Pol II at the gene body.^19,20^

In addition to regulating Pol II and transcriptional elongation, KMT2A-FPs also dysregulate self-renewal gene expression through epigenetic mechanisms. KMT2A-FPs associate with the Disruptor of Telomeric Silencing 1-Like (DOT1L) histone methyltransferase to catalyze transcription-activating histone H3 lysine 79 mono-, di-, and tri-methylation (H3K79me1/me2/me3).^9,21,22^ During normal hematopoiesis, MLLT3 and MLLT10 function as readers of H3K9 and H3K27 acetylation (H3K9ac, H3K27ac) and unmodified H3K27, respectively, both of which are associated with active transcription.^23,24^ Chromatin-bound MLLT3 and MLLT10 bind DOT1L and act as co-factors to promote DOT1L-mediated H3K79me2/me3 to active genes such as the *HOXA* cluster and maintain self-renewal gene expression programs in HSPCs.^22,23,25-27^ As these genes become inactivated during lineage commitment, marks read by MLLT3 and MLLT10 are lost and DOT1L dissociates from the chromatin, resulting in a switch to H3K79 mono-methylation, decreased HOX expression, and subsequent differentiation.^22,26,27^ In the context of MLL-r leukemia, the histone readers are fused to KMT2A and thus target DOT1L to *HOXA* cluster genes and *MEIS1* through the KMT2A-menin-LEDGF complex, resulting in H3K79 methylation and transcription activation at these loci.^14,22,26^

A key factor in the leukemogenicity of KMT2A-FPs is menin-mediated localization and binding of the KMT2A component to self-renewal loci. In this role as a mediator of KMT2A-chromatin binding, menin is required for both the initiation and maintenance of leukemic transformation driven by KMT2A rearrangements. In KMT2A-MLLT3 cells, loss of menin specifically reduces the expression of genes that are upregulated by the fusion protein, but KMT2A deletion did not have the same effect on these genes.^28,29^ Therefore, although menin is associated with both endogenous KMT2A and KMT2A-FPs, it appears to only be required for the function of the fusion protein in the context of AML. Chen et al. found that menin deletion specifically alters the expression of unique KMT2A-FP targets, which is likely related to a greater dependency on the N-terminal menin interaction following the loss of C-terminal chromatin targeting motifs in the fusion proteins.^29^ Aubrey et al. propose a role for the transcription factor IKZF1 as a regulator of the KMT2A-FP dependency on menin. They found that IKZF1 physically interacts with both menin and KMT2A at the transcription start sites (TSSs) of KMT2A-FP targets, and despite its function as a sequence-specific DNA-binding protein, it may direct the localization of menin-KMT2A-FP in a manner similar to LEDGF.^18,30^ Clearly, the interaction of KMT2A-FPs with menin is a critical mechanism of transformation and pathologic self-renewal in MLL-rearranged leukemias.

### NPM1-mutated AML

Nucleophosmin (NPM1) is a multifunctional protein with several different critical cellular functions, including ribosome biogenesis, DNA damage repair, and histone chaperoning. In this capacity, NPM1 facilitates cytoplasmic-nuclear transport of histones and controls histone deposition or removal from DNA. *NPM1* is among the most frequently mutated genes in AML (found in 30% of newly diagnosed patients), and *NPM1* mutations often co-occur with mutations in *FLT3* and *DNMT3A*.^31-33^ These mutations in NPM1 involve 4 base-pair insertions or duplications within the C-terminal three-helix bundle (3HB) and result in the loss of the nucleolar localization signal and the generation of a nuclear export signal (NES). As a result, the majority of the mutant protein, called NPM1c, (~90%) is mislocalized to the cytoplasm, a key feature of NPM1-mutant AML.^32,34-36^

Like in MLL-rearranged leukemias, aberrant expression of *HOX* genes and *MEIS1* in hematopoietic progenitor cells is a distinct feature of NPM1c AML.^36,37^ Recent studies propose that the nuclear exporter Chromosomal Maintenance 1 (CRM1) binds the mutant NES to direct residual nuclear NPM1c to these loci, where it cooperates with the wildtype KMT2A complex (which is present at these loci in healthy HSCs) to directly bind chromatin and inappropriately upregulate transcription in committed progenitors in a manner akin to that of KMT2A-fusion proteins (Figure 1A). While KMT2A does not appear to directly physically interact with NPM1c, menin-mediated wildtype KMT2A occupancy is a necessary prerequisite for the recruitment of NPM1c to the chromatin of leukemogenic genes, which may explain why NPM1c AML cells are sensitive to menin inhibition despite their lack of KMT2A-fusion proteins.^36-39^ NPM1c does, however, form multivalent heterotypic interactions with Pol II and components of the SEC and acts as a scaffold to stabilize the transcriptional machinery at its target genes. Consistent with this, degradation of NPM1c results in the loss of Pol II at the TSS and gene bodies as well as the loss of pTEFb binding to NPM1c target genes, indicating an important role for NPM1c in both transcription initiation and elongation.^36,37^

Chromatin immunoprecipitation (ChIP) studies further demonstrate that NPM1c binding not only activates gene expression by increasing transcriptional activity but also acts through epigenetic mechanisms resulting in the deposition and stabilization of activating histone marks. Histone acetylation, particularly H3K27ac and H3K9ac, is associated with active transcription, in part through recruitment of DOT1L-mediated H3K79me2/3 as described above. At the *HOXA* cluster and *MEIS1* loci, NPM1c antagonizes histone deacetylase (HDAC) activity to prevent the epigenetic silencing of those genes that normally occur during differentiation. In line with this HDAC-inhibitory function, NPM1c degradation results in the loss of H3K27ac and H3K9ac and subsequent loss of DOT1L, specifically at NPM1c binding targets, which results in decreased expression of self-renewal target genes.^36-38,40^ A similar effect may occur when NPM1c pentamerizes with wildtype NPM1 and displaces it from the nucleus, which may be another mechanism explaining how the NPM1c mutation dysregulates self-renewal gene expression.

Although NPM1c degradation does not disrupt KMT2A occupancy, NPM1c chromatin binding alone does not appear to be sufficient to activate the leukemogenic gene program, suggesting that the cooperation between KMT2A and NPM1c is likely more complex than just one recruiting the other, with NPM1c perhaps functioning analogous to the fusion protein components in MLL-r leukemias.^36^ Given that KMT2A binding normally regulates *HOX* and *MEIS1* gene expression in HSCs during healthy hematopoiesis, the recruitment of mutant NPM1c to those loci likely co-opts the KMT2A-associated chromatin regulators and inappropriately locks those genes into an active transcriptional state through stabilization of the transcriptional machinery and activating histone marks, thus preventing differentiation and maintaining cellular self-renewal.

### Targeting the KMT2A-complex through menin inhibition

#### Menin inhibitor monotherapy

Both MLL-r and NPM1c acute leukemias demonstrate a strong dependency on KMT2A complex-chromatin binding to maintain high leukemogenic gene expression and promote cellular self-renewal. Accordingly, the menin-KMT2A interaction is a targetable vulnerability in both AML subtypes. There are several ongoing clinical trials investigating the efficacy of small molecule inhibitors of the menin-KMT2A interaction for the treatment of relapsed/refractory MLL-r and NPM1-mutant AMLs (Table 1). The primary mechanism of current menin inhibitors is to prevent the localization and binding of KMT2A-FPs or wildtype KMT2A (and thus NPM1c) to their target genes, which results in (1) loss of KMT2A-mediated H3K4 methylation and DOT1L-mediated H3K79 methylation, (2) loss of binding to Pol II/SEC transcriptional machinery, and (3) derepression of HDAC activity, ultimately resulting in repression of *HOX* and *MEIS1* gene expression (Figure 1B and C).^36-38^ Additionally, dissociation of the KMT2A-complex from chromatin appears to promote menin ubiquitination and proteasome degradation, which likely destabilizes other KMT2A-complex components.^41,42^ As such, menin inhibitors primarily induce apoptosis and myeloid differentiation in preclinical AML models.^14,38,43-46^

**Table 1.** Clinical trials of menin-targeting mono- or combination therapy in the treatment of AML.

	Reference	Phase	Agent(s)	Type/Target	Genotype	Patient group + AML status	Status
**Monotherapy**	NCT04067336 (KO-MEN-001)	1/2	Ziftomenib (KO-539)	Menin inhibitor	MLL-r NPM1c	Adult R/R	Recruiting
	NCT04065399 (AUGMENT-101)	1/2	Revumenib (SNDX-5613)	Menin inhibitor	MLL-r NPM1c	Adult; child (>30 d) R/R	Recruiting, Approved for MLL-r leukemia
	NCT04811560 (CR108998)	1/2	JNJ-75276617	Menin inhibitor	MLL-r NPM1c NUP98/NUP214-m	Adult; child (>12 y) R/R	Recruiting
	NCT05153330 (COVALENT-101)	1	BMF-219	Menin inhibitor	MLL-r NPM1c	Adult R/R	Recruiting
	NCT06052813 (BN104-101)	1/2	BN-104	Menin inhibitor	MLL-r NPM1c	Adult R/R	Recruiting
	NCT04988555 (DSP-5336-101)	1/2	DSP-5336	Menin inhibitor	MLL-r NPM1c ± FLT3/IDH2-m	Adult; child (> 30d) R/R	Recruiting
**Combination therapy**	NCT05735184 (KO-MEN-007)	1	Ziftomenib (KO-539) + daunorubicin + cytarabine	Menin inhibitor + chemotherapy	MLL-r NPM1c	Adult new Dx	Recruiting
			Ziftomenib (KO-539) + venetoclax + azacitidine	Menin inhibitor + BCL2 inhibitor + hypomethylating agent	MLL-r NPM1c	Adult R/R	Recruiting
	NCT05886049 (NCI-2023-04141)	1	Revumenib (SNDX-5613) + daunorubicin + cytarabine	Menin inhibitor + chemotherapy	MLL-r NPM1c (no FLT3-m)	Adult new Dx (fit)	Recruiting
	NCT05326516 (AUGMENT-102)	1	Revumenib (SNDX-5613) + fludarabine + cytarabine	Menin inhibitor + chemotherapy	MLL-r or amp NPM1c NUP98-r	Adult; child (> 30d) R/R	Closed
	NCT05360160 (SAVE)	1/2	Revumenib (SNDX-5613) + ASTX727 + venetoclax	Menin inhibitor + hypomethylating agent + CDA inhibitor + BCL2 inhibitor	MLL-r NPM1c NUP98-r	Adult; child (> 12y) R/R; new Dx (unfit) AML/MPAL	Recruiting
	NCT06222580 (OSU-23199)	1	Revumenib (SNDX-5613) + gilteritinib	Menin inhibitor + FLT3 inhibitor	MLL-r + FLT3-m NPM1c + FLT3-m	Adult R/R	Recruiting
	NCT05453903 (CR109124)	1	JNJ-75276617 + daunorubicin + cytarabine	Menin inhibitor + chemotherapy	MLL-r NPM1c	Adult new Dx (fit)	Recruiting
			JNJ-75276617 + venetoclax + azacitidine	Menin inhibitor + BCL2 inhibitor + hypomethylating agent	MLL-r NPM1c	Adult R/R; new Dx (unfit)	

Abbreviations: ASTX727 = decitabine + cedazuridine; CDA = cytidine deaminase; Dx = diagnosis; MPAL = mixed phenotype acute leukemia; MTX = methotrexate; R/R = relapsed/refractory.

In KMT2A-MLLT3-fusion MOLM13 cells and NPM1-mutant OCI-AML3 cells, the menin inhibitors SNDX-50469 and ziftomenib (KO-539) inhibited proliferation and promoted differentiation, as evidenced by decreased *MEIS1* and *PBX3* expression with increased expression of the myeloid marker CD11b.^14,44,45,47^ Similarly, VTP50469 and the closely-related drug revumenib (SNDX-5613) significantly improved survival in xenograft mouse models of KMT2A-MLLT3 and NPM1-mutant AML.^14,45^ Early-phase clinical trials with ziftomenib (KO-MEN-001) and revumenib (AUGMENT-101) in patients with MLL-r or NPM1-mutant relapsed or refractory (R/R) AML show meaningful patient responses to menin inhibitor treatment. Recent  updates show that ~30% of patients achieve complete remission with or without complete hematologic recovery (CR/CRi) with a low incidence of severe treatment-related effects and adverse effects after treatment with either drug.^39,48-51^ Consistent with the differentiation phenotype observed in the preclinical studies, one of the most common treatment-related adverse effects in both trials was differentiation syndrome. Notably, NPM1c patients receiving 600 mg of ziftomenib who experienced differentiation syndrome had a higher overall response rate (75%) compared with the broader NPM1c treatment group (42%).^48^ Other menin inhibitors currently under clinical investigation, including JNJ-75276617 (NCT04811560) and BMF-219 (COVALENT-101), also appear to have similar antileukemic efficacy with tolerable safety profiles.^39,51-53^ The structures and mechanisms of the menin inhibitors listed in Table 1, as well as recent preclinical and clinical trial results, have been discussed extensively in other reviews.^13,39,42,51^

#### Mechanisms of resistance to menin inhibitors

Although clinical trials have shown that menin inhibitors are generally well-tolerated in patients and can induce remission with the elimination of minimal residual disease, the majority of patients do not respond to monotherapy and many relapse due to acquired missense resistance mutations in the menin gene (*MEN1*). These mutations frequently lead to changes in the G331, T349, and S160 amino acids of the menin-binding pocket that interfere with menin-inhibitor binding or decrease inhibitor avidity. Importantly, these mutations only disrupt the binding of small molecules, but not KMT2A, to menin, permitting sustained oncogenic activity with resistance to menin inhibition.^54^ Alternative indirect mechanisms of menin inhibitor activity have been proposed recently that suggest that these inhibitors not only repress expression of leukemogenic genes but also promote the expression of tumor suppressor genes that are involved in cell-cycle arrest and senescence through a mechanism known as the menin-UTX “switch.”^55^ For a unique set of genes with tumor suppressor functions, separate from canonical KMT2A-FP targets, menin-KMT2A binds to promoters to maintain transcriptional repression. Upon menin inhibitor treatment, the menin-KMT2A complex is displaced from the promoters of these genes which include cyclin-dependent kinase (CDK) inhibitors and are replaced by the KMT2C/D-UTX complex. Resultant KMT2C/D-mediated H3K4me1, UTX-mediated H3K27 de-methylation, and H3K27ac result in the activation of genes involved in cell-cycle arrest and senescence.^55,56^ Acquired UTX loss-of-function mutations are common in AML and are associated with resistance to chemotherapy (eg, cytarabine and daunorubicin) as well as disease relapse.^57-60^ Importantly, menin inhibitor-induced differentiation and loss of *HOX/MEIS1* expression do not inhibit leukemic cell proliferation in the context of UTX loss, suggesting that loss of this menin-UTX “switch” mechanism and failed co-induction of tumor-suppressive programs are similarly implicated in resistance to menin inhibition.^55^

Another purported mechanism of menin inhibitor resistance is through DOT1L, which is recruited to leukemogenic loci by the fusion partner components of KMT2A-FPs. Disruption of this oncogenic complex pushes cells to maximize native DOT1L activity for H3K79 methylation and overcome any loss of H3K79me2/3 that occurred after menin inhibition.^26,61^

Despite the emergence of resistance mechanisms to menin inhibition, the success of these drugs in early clinical trials supports the investigation of other menin-KMT2A complex cooperators as potential synergistic targets to overcome menin inhibitor resistance and associated relapse.

### Combinatory targeting of menin and other KMT2A-related chromatin regulators

#### Current combination therapies in clinical trials

To improve the efficacy and sustainability of menin inhibitor effects, there have been several clinical trials investigating combinations of these drugs with standard AML treatments such as chemotherapy (KO-MEN-007, NCI-2023-04141, AUGMENT-102, CR109124), venetoclax with hypomethylating agents (KO-MEN-007, CR109124, SAVE), or FLT3 inhibitors in the case of co-existing FLT3 mutations (OSU-23199) (Table 1).^45,46,62-66^ Although results in the early stages of these trials appear promising, it may be necessary for new therapeutic strategies to focus on targeting both menin and proteins that co-regulate the chromatin landscape in conjunction with the menin-KMT2A complex to overcome the current challenges associated with menin inhibitor resistance and AML relapse.

#### DOT1L inhibitors

As discussed above, KMT2A-FP and the wildtype fusion partners (MLLT3, MLLT10) recruit DOT1L to promote H3K79me2 at *HOX* and *MEIS1* chromatin and activate their transcription in both MLL-r and NPM1c AML.^67^ There is evidence that DOT1L function is not only dysregulated in AML cells but also necessary for their viability, supporting the investigation of DOT1L as a therapeutic target, although H3K79 methylation itself does not appear to be critical for leukemogenesis.^38,68^ However, Gilan et al. showed that there is not the same requirement for H3K79me2 in maintaining leukemic cell viability, which they attribute to the existence of 2 functionally separate complexes in these cells: an oncogenic DOT1L-KMT2A fusion complex, and a native DOT1L-MLLT10 complex.^26^ Disruption of either complex by DOT1L inhibitors appears to result in DOT1L redistribution and increased association with the other, thus resulting in enrichment of H3K79me2 at specific complex targets despite a global depletion of H3K79 methylation. Furthermore, inhibition of menin by VTP50469 (and thus loss of KMT2A-mediated DOT1L localization) does not result in the loss of H3K79me2 at all KMT2A-MLLT3 targets, due to the continued function of the native DOT1L complex at those loci. This suggests that the disruption of the native DOT1L complex would increase sensitivity to the disruption of the oncogenic DOT1L complex by menin inhibitors (Figure 1B).^26,61^

Indeed, several preclinical studies found that the combination of menin and DOT1L inhibition displaces KMT2A-FPs from chromatin more quickly than single-agent treatment, and H3K79me2/3 at *HOX* and *MEIS* loci is decreased far more significantly after combination treatment in both MLL-r and NPM1c AML. Leftward shifts in dose–response curves in preclinical models demonstrate synergy between the following combinations of DOT1L and menin inhibitors: SGC0946 with MI-503 or MI-2-2, and EPZ5676 or EPZ004777 with MI-2-2.^26,38,40,61,69,70^ This effect is likely explained by the fact that DOT1L inhibitors are not specific for KMT2A-FP-bound DOT1L and thus target both oncogenic and native H3K79 methylation. There are currently no open clinical trials of DOT1L inhibitors, and completed trials of pimenostat found that the drug was well-tolerated and safe but demonstrated modest efficacy as a single agent, with only a limited number of patients achieving full remission (Table 2).^71^ Still, the established safety profile of this inhibitor in addition to preclinical evidence that DOT1L inhibitors increase sensitivity to menin inhibition supports future trials investigating combined menin and DOT1L inhibition as an effective strategy to overcome menin inhibitor resistance.

**Table 2.** Clinical trials targeting PRC1/2 and KMT2A-related chromatin regulators in the treatment of AML.

	Reference	Phase	Agent(s)	Type/Target	Genotype	Patient group + AML Status	Status
**DOT1L**	NCT01684150 (EPZ-5676-12-001)	1	Pinometostat (EPZ-5676)	DOT1L inhibitor	MLL-r KMT2A-PTD	Adult R/R	Completed
	NCT02141828 (EPZ-5676-12-002)	1	Pinometostat (EPZ-5676)	DOT1L inhibitor	MLL-r	Child (>3m) R/R	Completed
	NCT03701295 (NCI-2018-02128)	1/2	Pinometostat (EPZ-5676) + azacitidine	DOT1L inhibitor + hypomethylating agent	MLL-r	Adult R/R; new Dx (fit/unfit)	Completed
	NCT03724084 (NCI-2018-02349)	1/2	Pinometostat (EPZ-5676) + daunorubicin + cytarabine	DOT1L inhibitor + chemotherapy	MLL-r	Adult new Dx (fit)	Terminated
**CDK4/6**	NCT02310243 (AMLSG 23-14)	1/2	Palbociclib	CDK4/6 inhibitor	MLL-r	Adult R/R; new Dx (unfit)	Unknown
	NCT03132454 (2016-0772)	1	Palbociclib + sorafenib	CDK4/6 inhibitor + chemotherapy	Unspecified	Adult R/R	Recruiting
			Palbociclib + decitabine	CDK4/6 inhibitor + hypomethylating agent	Unspecified	Adult R/R	
			Palbociclib + dexamethasone	CDK4/6 inhibitor + glucocorticoid	Unspecified	Adult R/R	
			Palbociclib + venetoclax	CDK4/6 inhibitor + BCL2 inhibitor	Unspecified	Adult R/R	
	NCT03844997 (CASE1918)	1/2	Palbociclib + CPX-351	CDK4/6 inhibitor + chemotherapy	Unspecified	Adult new Dx; therapy-related; 2/2 MDS	Recruiting
	NCT05627232 (22G.769)	1	Palbociclib + CPX-351	CDK4/6 inhibitor + chemotherapy	Unspecified	Adult R/R	Recruiting
**PRC1/2**			Tazemetostat + CPX-351	EZH2 inhibitor + chemotherapy	Unspecified	Adult R/R	Recruiting
	NCT03110354 (DS3201-A-U102)	1	Valemetostat (DS-3201b)	EZH1/EZH2 dual inhibitor	Unspecified	Adult R/R	Terminated

Abbreviations: CPX-351 = daunorubicin + cytarabine; Dx = diagnosis; MDS = myelodysplastic syndrome; R/R = relapsed/refractory.

#### CDK4/6 inhibitors

UTX-induced expression of tumor-suppressive programs, including genes associated with cell-cycle arrest and therapy-induced senescence, is necessary for the antileukemic effects of menin inhibitors, and loss of UTX function is implicated in menin inhibitor resistance.^55^ Upon menin inhibition, UTX directly upregulates the expression of *Cdkn2c/Ink4c* and *Cdkn2d/Ink4d*, which encode inhibitors of the CDKs—CDK4 and CDK6. Menin inhibitor/UTX-induced expression of these genes results in decreased CDK4/6 expression and cell-cycle arrest, which is lost upon UTX deletion. Soto-Feliciano et al. found that preclinical treatment of KMT2A-MLLT3 cells with the FDA-approved CDK4/6 inhibitor palbociclib could rescue cell-cycle arrest in the context of UTX deletion and restore sensitivity to menin inhibition.^55^ Consistent with this, combinations of menin inhibitors with palbociclib or other CDK4/6 inhibitors abemaciclib or ribociclib can promote synergistic lethality in both MLL-r and NPM1c AML models but not in healthy HSPCs.^45,66^ Although CDK6 and CDK4 are thought to act redundantly to promote cell-cycle progression, there is evidence that MLL-r leukemias are also dependent on the noncanonical antidifferentiation functions that are specific to CDK6, which may be mediated by direct KMT2A-FP up-regulation of CDK6 expression.^72-74^ This suggests that CDK6 inhibition may improve the response to menin inhibitors by promoting not only cell-cycle arrest and apoptosis but also through promoting myeloid differentiation (Figure 1C).

Palbociclib, abemaciclib, and ribociclib are all approved by the FDA for the treatment of breast cancer. There are also several ongoing clinical trials investigating palbociclib as a single agent or in combination with standard chemotherapy for the treatment of AML (Table 2). This drug appears to be generally well-tolerated in hematologic malignancies, although the efficacy of the single agent seems to be minimal in the context of relapse/refractory MLL-r AML.^75-77^ Still, the preclinical evidence suggests that combinatory treatment with menin and CDK4/6 inhibitors may be more effective in AML than monotherapy and supports the initiation of future studies exploring the potential of these combinations in improving patient clinical outcomes.

## Polycomb repressive complexes in AML

### Mechanisms of PRC2 and PRC1 regulation of gene expression in AML

The polycomb repressive complexes 1 and 2 (PRC1/2) are 2 distinct protein complexes involved in depositing histone marks associated with gene repression. In healthy cells, PRC2 deposits the repressive tri-methylation mark at H3K27 (H3K27me3), which recruits PRC1 to deposit the repressive mono-ubiquitination mark on H2AK119. Both complexes have been implicated in AML.

Canonical PRC2 is comprised of 4 key subunits: EZH1 or EZH2 (catalytic histone methyltransferase), SUZ12, EED, and RBBP4/7 (histone binding). The functional and structural roles of PRC2 have been beautifully reviewed recently by Bhattacharyya and Bond.^78^ In their review, they highlight a strong prognostic link between PRC2 alterations and negative therapeutic outcomes, with decreases in PRC2 function correlating strongly with more aggressive AML and a poorer prognosis.

**Figure 2.**
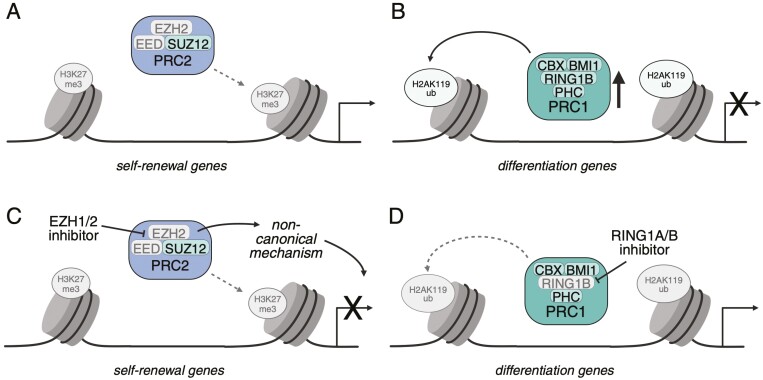
(A) PRC2 has reduced function in AML, contributing to decreased H3K27me3, especially at self-renewal loci. (B) PRC1 is hijacked in AML to enhance the repression of differentiation-associated genes by increased levels of H2AK119ub. (C) Targeting EZH1/2 and PRC2 impairs leukemic self-renewal, likely by non-canonical EZH2 function. (D) RING1A/B and PRC1 inhibition push leukemic cells to differentiate. Figure created with BioRender.com

In AML, PRC2 functions are perturbed through a variety of mutational mechanisms. Mutations in each of the core components of PRC2 have been identified in AML cases.^78^ The most common are EZH2 mutations, typically found within the SET domain that is responsible for EZH2 catalytic activity. These mutations result in decreased global H3K27me3, which underscores the role that the polycomb proteins play in regulating genes involved in stem cell self-renewal and hematopoietic lineage commitment^79^ (Figure 2A). Many groups have studied the roles of the PRC2 components, especially EZH2 and EED, with contradictory findings regarding PRC2 activity in mouse models of AML; some groups identified PRC2 as necessary for leukemogenicity, while others found that the loss of PRC2 components confer enhanced leukemic capacity.^80-82^ A seminal study by Basheer et al. elucidated these contradictory findings and attributed these to the disease stage.^83^ They discovered that EZH2 and PRC2 play an oncogenic role at disease onset and a tumor-suppressive role during disease maintenance. Basheer et al. uncovered that this difference is primarily due to the derepression of distinct gene programs at disease onset compared with disease maintenance. Specifically, loss of H3K27me at bivalent promoters contributes to enhanced oncogenicity during AML induction, while the same loci remain unaltered during disease maintenance upon targeting EZH2.^83^ This highlights the importance of understanding the disease stage when designing treatment regimens.

Mutations in other PRC2 components have also been implicated in AML.^78^ EED and RBBP4/7 are primarily mutated in the WD domains that help facilitate the complex interactions, and SUZ12 mutations have been discovered in the zinc-binding factor domain, which facilitates interactions with additional proteins associated with PRC2. Although these mutations have distinct biochemical implications, the overall functional readout is diminished PRC2 activity, as marked by a decrease in global H3K27me3.

Outside of the core components, a variety of additional subunits can regulate PRC2 activity. ASXL1, a chaperone protein involved in guiding PRC2 to its target loci, is among the most frequently mutated genes in both myelodysplastic syndrome (MDS) and AML.^78,84,85^ Mutations in ASXL1 result in a truncated protein, reducing its ability to guide PRC2 to its target loci and resulting in a global reduction in H3K27me3 levels.^84^ As expected, genetic inactivation of *Asxl1* in mouse hematopoietic cells leads to aberrant self-renewal and impaired differentiation.^84,85^

PRC1 is also gaining interest in the field as a druggable target in AML and can be sorted into 2 separate subcategories: canonical PRC1 and noncanonical PRC1.^86^ All iterations of PRC1 contain RING1 and PCGF proteins. In canonical PRC1, CBX and PHC units join the complex, whereas they are not incorporated into noncanonical PRC1 variants. These variants are responsible for the deposition of the H2A mono-ubiquitination, whereas the canonical PRC1 is more involved in chromatin compaction and looping.^86^ A key component of the canonical PRC1 complex is the PCGF protein BMI1, which fuels the E3 ligase activity of the RING proteins in the deposition of H2A mono-ubiquitination.^87^ PRC1 function is hijacked in AML to repress differentiation-associated programs, contributing to enhanced self-renewal of the leukemic cells^88^ (Figure 2B). Like EZH2, PRC1 functions in AML to regulate bivalent loci and keep key differentiation genes silenced, which contributes to the maintenance of PRC2 repression at these loci as well.^88^ This has provided the rationale for inhibition of PRC1 to differentiate leukemic cells, with varying levels of success, as discussed below.

The eraser of the mark deposited by PRC1 is the BAP1-deubiquitinase complex. In ASXL1-mutated cases of AML, the truncated ASXL1 protein gains a new function in which it cooperates with the BAP1 complex to promote enhanced deubiquitination.^89,90^ This further highlights the complex relationships between the PRC complexes and specific mutations.

### Targeting polycomb repressive complexes in AML

#### Targeting PRC2 in AML

As previously explained, elucidating the role of EZH2 in AML has been a complex journey. Given these context-dependent complexities, it is not surprising that the targeting of EZH2 and EZH1/2 in preclinical studies has had somewhat mixed results. Most notably, Fujita et al. found that the joint targeting of both EZH1 and EZH2 necessary to impair leukemic cell proliferation. They also found that dual targeting of EZH1 and EZH2 is better able to deplete the LSC pool compared with targeting EZH2 alone.^91^ This is likely due to known compensation mechanisms between EZH2 and EZH1 in catalyzing H3K27 tri-methylation in hematopoietic cells.^92,93^ These preclinical data led to the use of the dual EZH1/2 inhibitor valemetostat as a single agent in a Phase 1 clinical trial in AML and ALL (NCT03110354, Table 2), but this trial was terminated prematurely due to slow enrollment. None of the enrolled patients completed the study, primarily due to the lack of response or disease progression. Although at face value it appears that valemetostat does not have clinical promise, these trial results suggest that this class of drug is not sufficient as a single agent and may be more effective in combination with other therapies.

Somewhat counterintuitively, Alqazzaz and colleagues discovered that EZH2 LOF patient samples are more sensitive to EZH2 and PRC2 inhibition compared with wildtype EZH2 samples^94^ (Figure 2C). This is especially enlightening as multiple groups have now discovered decreases in EZH2 protein levels to be a nongenetic resistance mechanism to some AML therapies, including chemotherapy and FLT3 inhibition, indicating that combination with EZH2/PRC2 inhibitors may be beneficial.^95,96^ This also indicates that EZH2 may have noncanonical functions in the leukemic setting beyond its histone methyltransferase activity (Figure 2C). Consistent with this idea, Velez et al. found that an EZH2 PROTAC degrader induces enhanced EZH2 degradation and has stronger antiproliferative effects than other methods to target EZH2 in AML cells.^97^

In studying therapeutic vulnerabilities in ASXL1-mutant settings, Ge and colleagues have identified KDM6B, the histone demethylase responsible for the removal of H3K27me3 deposited by PRC2, as a promising target.^98^ Treatment with the KDM6B inhibitor GSK-J4 was able to restore global H3K27me3 levels in leukemic NSG mice, leading to reduced transcriptional activity of aberrantly activated genes in ASXL1-mutant cells and reduced leukemic capacity.^98^ As targeting the components of PRC2 has proven to be difficult, especially in the context of ASXL1 mutations, this strategy is an elegant workaround to impact global H3K27me3 levels and was demonstrated to prevent leukemic transformation caused by ASXL1 mutations. Although these findings are preclinical, the results support this strategy moving to clinical trials soon.

#### Targeting PRC1 in AML

In exploring therapeutic options for targeting PRC1, BMI1 was uncovered as an attractive target when Rizo et al. discovered that knocking down BMI1 impairs the self-renewal capacity of leukemic stem cells.^99^ Additionally, Shima et al. have shown that RING1A/B activity is necessary for leukemogenesis and disease progression in mouse models of AML.^88^ To this end, Shukla et al. sought to design small molecule inhibitors targeting PRC1. They successfully designed a first-in-class small molecule inhibitor (RB-3) targeting RING1B and RING1A, leading to inhibition of PRC1 activity and global reductions in H2AK119ub. This subsequently pushes leukemic cells to differentiate^100^ (Figure 2D). While this finding offers exciting possibilities for PRC1 interventions, the specific molecular characteristics that makes leukemic cells susceptible to this treatment remain to be determined.

ASXL1 is also associated with modulating PRC1 activity, where mutant ASXL1 has enhanced cooperativity with the BAP1 deubiquitinase complex, which in turn leads to an aberrant global reduction of H2AK119ub.^89,90,101^ This interaction has prompted numerous groups to focus on components of PRC1 as therapeutic targets. Recent work from Sparbier et al. has highlighted the intricate interplay between KMT2A/B and polycomb that is necessary for proper regulation of HSC stemness and differentiation genes.^102^ Using whole-genome CRISPR-Cas9 screens in conjunction with MHC class I gene expression, they found that menin inhibition phenocopies polycomb disruption. MHC class I gene expression is regulated via bivalent histone modifications during development, which allowed them to identify the polycomb disruption specifically at bivalent loci (containing repressive and activating marks) that are unique to stem cells. Inactivation of PRC1 is also implicated in menin inhibitor resistance, which similarly results in the inhibition of tumor-suppressive programs through the loss of H3K27 methylation, the loss of CDK inhibitor expression, and the overactivation of MYC.^103,104^ Therefore, there may be a rationale for combining menin inhibitors with EZH2/PRC2 or PRC1 inhibitors.

## Conclusions

Multiple components of the epigenetic machinery, including those that catalyze histone modifications, have emerged as key therapeutic targets in AML. Understanding how histone writers such as KMT2A and PRC1/2 interact with transcriptional machinery and other chromatin regulators to contribute to leukemic transformation as well as to therapeutic resistance and disease relapse is critical for the development of novel strategies to target aberrant epigenetic regulation. Over the last decade, there has been tremendous progress in developing therapies targeting KMT2A activity through menin inhibition, and there is mounting evidence to suggest that these drugs may synergize with inhibitors of other proteins in the extended KMT2A network such as DOT1L and EZH2 to increase the sensitivity to menin inhibition and overcome drug resistance. Many inhibitors of these other interactors are already being explored in clinical trials, and these studies provide a rationale for further clinical investigation of menin inhibitors in combination with other epigenetic therapies—even those that appear to have minimal efficacy as single agents—to determine if the preclinical evidence discussed in this review can be translated more effective means of treating relapsed and refractory AML. Targeting PRC2 and PRC1 has proven more difficult in AML, given the mostly inactivating mutations observed in members of these complexes in AML, as well as the varying dependencies on PRC2 at different stages of AML progression. However, it is possible that PRC1/2 inhibitors will prove to be useful in combination treatment, including in combination with menin inhibitors.

## Data Availability

No new data were generated or analyzed in support of this research.
